# Supplementary material for: What is the relationship between validated frailty scores and mortality for adults with COVID-19 in acute hospital care? A systematic review
Source: Age Ageing. 2021 Jan 14;50(3):608–16. doi: 10.1093/ageing/afab008 (PMC7929406; doi:10.1093/ageing/afab008)
Supplement: aa-20-1566-File002_afab008 [file aa-20-1566-file002_afab008.docx]

# What is the relationship between validated frailty scores and mortality for adults with COVID-19 in acute hospital care? A systematic review.

**SUPPLEMENTARY DATA**

*Appendix 1 Grading of papers using the NOS scale*
